# Complete Agenesis of Dorsal Wall of Sacral Canal: A Case Report

**DOI:** 10.7759/cureus.5720

**Published:** 2019-09-21

**Authors:** Manisha Gaikwad, Babita Kujur, Mantu Jain, Sudhanshu S Das, Sudarsan Behera

**Affiliations:** 1 Anatomy, All India Institute of Medical Sciences, Bhubaneswar, IND; 2 Orthopaedics, All India Institute of Medical Sciences, Bhubaneswar, IND

**Keywords:** sacral canal, agenesis, caudal block

## Abstract

The sacral canal is the continuation of the vertebral canal in the sacrum. The sacral canal contains spinal meninges, lumbar and sacral part of spinal nerves and filum terminale. So sacral canal has been used for the caudal epidural block. During routine osteology demonstration classes for undergraduate students, we observed a dry human sacrum with complete agenesis of the dorsal wall of the sacral canal. Knowledge of these variations is important to diagnose lower back pain, sciatica, caudal regression syndrome and to avoid complications related to caudal epidural block and other spinal surgeries like in placement of screw for spinal fusion.

## Introduction

The sacral canal (SC) is the continuation of the vertebral canal in the sacrum. The SC is formed anteriorly by the fusion of sacral vertebral bodies and posteriorly is formed by the fusion of posterior elements namely the lamina, and spinous process [1]. It contains spinal meninges, lumbar and sacral part of spinal nerves and filum terminale. Clinically, the SC has been employed for administering the caudal epidural block (CEB). The CEB is an important tool for spinal surgeons and the domains of pain management as it is used to ameliorate the pain associated with lumbar and sacral nerve roots in a variety of lumbar spinal disorders. The benefit of a successful CEB has been reported to be as high as 70%-80% in the literature [2]. Though there are some of the identified cause for failure of procedure that may depend on the anatomical variation of SC especially in relation to sacral hiatus. With the advent of imaging techniques like ultrasonography or fluoroscopy, the hurdles faced during caudal epidural block due to anatomical variations can be overcome. But it is not always feasible due to time, cost and personal availability, so knowledge of these anatomical variations of the sacral hiatus or canal will facilitate the smooth performance of this procedure and decrease their failure rate.

Spina bifida occulta (SBO) is one of the situations, representing an open neural arch due to insufficient fusion of the posterior elements of the spine. Despite the frequent appearance of SBO in clinical practice, controversy exists as to its importance. Kettler and Wilke categorized the SBO defects on a scale from 0 to III depending on the percentage of opening in the posterior neural arch. Beginning with a normal state, Grade 0 being the unaffected complete posterior arch to grade III “pan-sacral” was defined as the failure of closure from S1-S5 [3].

We report a case of pan sacral agenesis of the dorsal sacral canal and discuss its clinical implications.

## Case presentation

**Figure 1:**
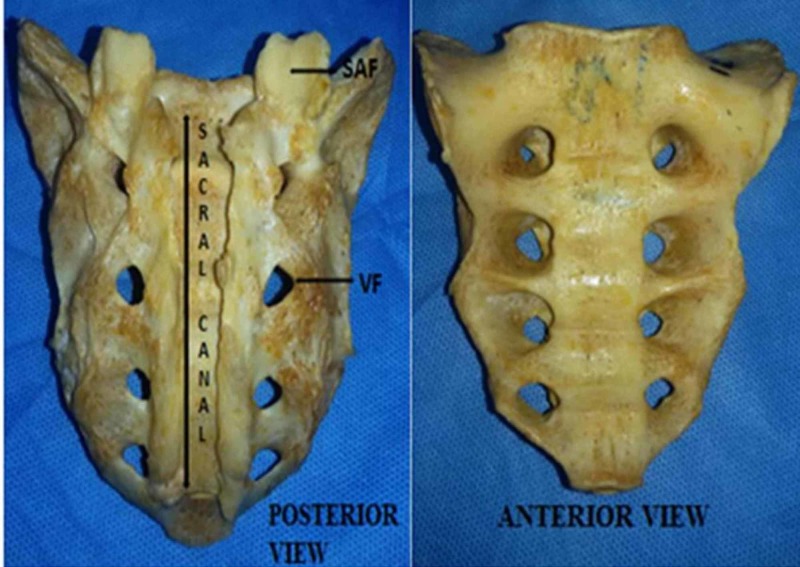
Photograph showing the posterior and anterior view of the sacrum SAF: Superior articular facet; VF: Vertebral foramen.

During routine osteology illustration classes for undergraduate students of anatomy department at our institute, we observed a dry human sacrum with complete agenesis of the dorsal wall of the sacral canal. The bone probably belonged to a male cadaver as the sacral index and other features were suggestive. The laminae of sacral vertebrae were fused laterally but open in the midline. Other findings like an intermediate sacral crest, which represents the fused articular processes, and lateral sacral crest, which represent the fused transverse process, were present normally. The dorsal sacral foramen (eight in number through which exits the dorsal rami of sacral nerves) were situated lateral to the intermediate crest (Figure 1).

**Figure 2:**
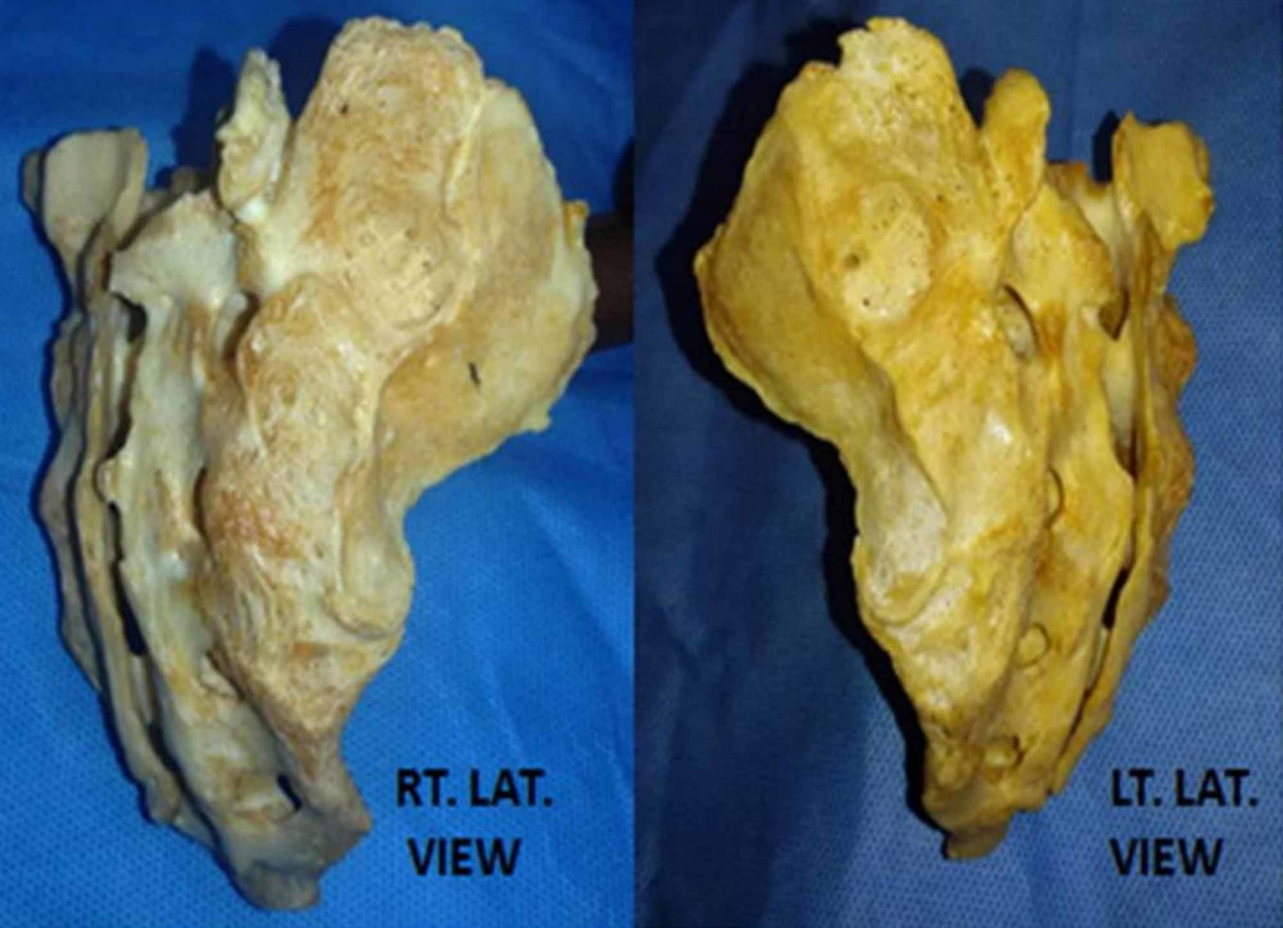
Photograph showing the right and left lateral view of the sacrum

The ventral and the lateral surface of the sacrum showed absolutely normal features without any anomaly (Figure 2).

## Discussion

Morphological variation of sacral hiatus like its shape, size, etc. has been reported several times in the literature. Among these variations, Hiatal agenesis and complete agenesis of the dorsal wall is a scarce finding. According to the literature, the incidence of pan sacral agenesis of SC ranges from 0.98% to 4.8% [2-4]. This is due to failure of fusion of the lamina of the sacral vertebrae to form the median sacral crest. Embryological basis of this condition is still not clear and assumed to be multifactorial. One of the clarifications has been the faulty induction of vertebra formation by the underlying notochord during embryological development, possibly by altering sonic hedgehog signaling [5]. Vertebrae are developed from somites which are divided into two parts - ventromedial sclerotome and dorsolateral dermomyotome. The vertebrae are formed from the sclerotome part of the somites. Spina bifida occulta occurs as a fallacy in tissue separation during secondary neutralization resulting in dorsal nonunion of the laminae [6]. In our case laminae of all the five sacral vertebrae were present but it has failed to fuse in the midline resulting in such defect. Though this can happen at any spinal level but commonly affects the lumbosacral region from L3 to S1 [6]. But in our case, there was complete agenesis of the dorsal wall of the sacral canal. Genetic causes of these defects remain elusive although according to Langman factors responsible for this condition are a mutation in VANGL gene and HOX gene [7]. VANGL genes are part of the planar cell polarity pathway that regulates convergent extension, the process that lengthens the neural tube and is necessary for normal closure to occur. Homeobox gene code for transcription factors activates the cascades of genes regulating phenomena such as segmentation and axis formation [7].

Several authors have reported this condition but its clinical significance remains contentious. At the one end of the spectrum lies complete asymptomatic variant and to the other end having a host of serious deficits including backache, lower limb and bladder/bowel affections [8]. Researchers have noted an interconnection between these lumbosacral vertebral anomalies and the alimentary canal malformations. However, the explanations remain putative because of poor evidence. Padmanabhan experimentally found that retinoic acid treatment of pregnant mice can induce caudal vertebral defects, spinal bifida, and imperforate anus [9]. We also have seen sacral vertebral defects in children with the anorectal abnormality. About a third of patients with anorectal anomalies have been recognized to have sacral malformation by ultrasonography or magnetic resonance imaging scans [5]. SBO was almost eight times as common in males compared to the females [10]. Our sacrum also seemed to belong to a male cadaver as suggestive by sacral index (95) and other features of sexual dimorphism of the sacrum.

CEB has been extensively used both for diagnostic and therapeutic management of lumbosacral disorders. The CEB involves passing a spinal needle through the sacral hiatus to distribute medications into the sacral epidural space. In case of pan sacral agenesis of the dorsal wall of sacral canal, the CEB is possible but this procedure becomes risky to complications like an accidental puncture of dura mater. Similarly, the spine surgeons may face difficulty in the exposure of the sacrum for the surgical procedure including instrumentation using the transpedicular screw for spinal fusion. Dorsal agenesis of SC also places the sacral spinal nerves to risk to injury during such internal screw fixation [4].

## Conclusions

Complete agenesis of the dorsal wall of the sacral canal of these variations is important to diagnose lower back pain, sciatica, caudal regression syndrome and to avoid complications related to caudal epidural block and other spinal surgeries like in placement of screw for spinal fusion.
